# An integrated model: marital effect on adolescent behavioral problems through siblings

**DOI:** 10.3389/fpsyg.2023.1282092

**Published:** 2024-01-08

**Authors:** Zhaoyi Li, Yunyan Zhao, Ran He, Rui Luo, Yuhan Luo, Zhengqian Yang, Mengdi Qi, Fumei Chen

**Affiliations:** ^1^Collaborative Innovation Center of Assessment Toward Basic Education Quality, Beijing Normal University, Beijing, China; ^2^Department of Psychology, University of Notre Dame, Notre Dame, IN, United States; ^3^State Key Laboratory of Cognitive Neuroscience and Learning, Beijing Normal University, Beijing, China; ^4^School of Education, Minzu University of China, Beijing, China

**Keywords:** marital relationship, sibling relationship, depressive symptoms, aggressive behavior, adolescent

## Abstract

**Introduction:**

Few studies have simultaneously focused on the effects of marital conflict and marital intimacy on adolescent development, and little is known about the role of sibling relationships. Thus, this study examined the association between marital relationships and adolescent behavioral problems, including depressive symptoms and aggressive behavior. At the same time, we explored the mediating role of sibling hostility and sibling affection and the moderating effect of birth order in multichild families in China.

**Methods:**

Participants included 842 adolescents (*M*age = 12.60, 46.2% boys) from Henan Province. Marital relationship, sibling relationship, birth order, depressive symptoms and aggressive behavior were assessed by a self-administered questionnaire. SEM was then used to examine the role of sibling relationships and birth order in the association between marital relationship and adolescent behavioral problems.

**Results:**

Our results showed that marital intimacy was negatively correlated with depressive symptoms and aggressive behavior, while marital conflict was positively correlated with them. Marital intimacy was associated with depressive symptoms and aggressive behavior through both sibling hostility and sibling affection. Marital conflict was indirectly associated with depressive symptoms and aggressive behavior through sibling hostility. In addition, the first-born adolescents were more sensitive to marital intimacy.

**Discussion:**

Given that the occurrence of adolescent behavioral problems is more common in contemporary society, our findings suggest that establishing a more intimate and warmer family atmosphere and promoting positive interactions between siblings may help control adolescent mental health problems.

## 1 Introduction

Adolescence is a critical time for the onset and development of behavioral problems, including internalizing problems, such as depressive symptoms, and externalizing problems, such as aggressive behavior (Blain-Arcaro and Vaillancourt, 2017; Murray et al., 2022). Depressive symptoms and aggressive behavior are two of the most common mental health difficulties in adolescents that often co-occur (Wolff and Ollendick, 2006; Speyer et al., 2022). Moreover, they may contribute to poor academic performance, problematic substance use, and even suicidality (Erath et al., 2006; Kraft et al., 2023). Accordingly, exploring factors that may decrease adolescent depressive symptoms and aggressive behavior has important implications.

According to ecological systems theory (Bronfenbrenner, 2000), family is the most important microsystem for children’s development, and the marital relationship has a strong effect on the function of the entire family. Marital relationships involve marital conflict and marital intimacy, two distinct constructs that do not represent opposing ends of the same continuum (McHale et al., 2000; Chen and Shi, 2017). Both are closely linked to adolescent development. For example, marital conflict may damage adolescent mental health and creates behavioral problems, including anxiety, depressive symptoms and elevated aggression (Philbrook et al., 2018; Li et al., 2020; Swerbenski et al., 2023). While decreased marital intimacy is related to decreased adolescent internalizing and externalizing problem behaviors over time (Chen and Shi, 2017; Knopp et al., 2017; Zhang et al., 2022), which is more important for cultivating adolescents’ positive qualities from the view of positive psychology. Nevertheless, in contrast to the extensive body of literature on marital conflict, there has been relatively limited focus on the significance of marital intimacy. How marital intimacy effect adolescent depressive symptoms and aggressive behavior needs to be further explored.

In addition to the parental relationship, the interaction with siblings holds significance in families with multiple children. Sibling relationships are usually among the earliest and longest lasting relationships formed in life, and adolescents spend more time interacting with siblings than with any other family member and peers (Cicirelli, 1985; McHale et al., 2012). As a love-hate relationship, it includes two dimensions: sibling hostility and sibling affection (Ruff et al., 2018; Zhao et al., 2021). Family systems theory emphasizes that family consists of multiple subsystems, such as parent-child subsystem and sibling subsystem, the subsystems are interdependent and influence each other (Minuchin, 1985). On the one hand, family spillover hypothesis suggests that stress or harmony from parents’ marriage may spill over into sibling relationships. Increases in marital conflict is related to increase in hostility in sibling relationships (Merino et al., 2022; Chen, 2023). Meanwhile, marital intimacy is positively related to sibling closeness (Kim et al., 2007), and adolescents with warm marital relationships have higher sibling affection (McHale et al., 2000; Skinner and McHale, 2022). Thus, marital conflict and intimacy may impact sibling hostility and affection. On the other hand, both warm and conflictual sibling interactions play significant roles in adolescent development (Li et al., 2019; Skinner and McHale, 2022). More specifically, sibling hostility is positively associated with higher levels of depressive symptoms (Campione-Barr et al., 2013), difficulties with peers, problems at school, and substance use (Odudu et al., 2020). At the same time, siblings are an important source of emotional support (Johnson and Salafia, 2022), and sibling affection is not only positively correlated with adolescents’ satisfaction with life and emotional wellbeing (Padilla-Walker et al., 2010; Harper et al., 2016) but also associated with lower instances of behavioral problems, including depressive symptoms and aggression (Branje et al., 2004; van Dijk et al., 2022). Thus, sibling relationships may mediate the connection between marital relationships and adolescent behavioral problems.

However, even within the same family, the effects of marital and sibling relationships on adolescent development are inconsistent. Concerning sibling difference, an important contributing factor is the birth order, which is defined as a person’s rank by age among his or her brothers and sisters (Volling, 2012). Resource depletion theory focus on resource depletion as a pathway through which children of lower birth orders may receive increased resources and investment compared to subsequent siblings (Stannard et al., 2019). While other studies holds that first-born children’s security may be threatened by disruptions to normal family life, and they may thus exhibit worry, anxiety, depressive symptoms and aggressive behavior more easily than second-born children (Buhrmester and Furman, 1990; Chen and Volling, 2023). And from Adler’s ethological/analytic perspective, parents’ tendencies to overindulge younger siblings is linked to the dethronement of the first-born, who is then more sensitive to the dynamics of the marital relationship, so marital conflict or intimacy may have a great impact on eldest siblings (McHale et al., 2012). Regarding sibling relationships, later-born adolescents have reported more companionship and affection with siblings (Volling, 2012; van Berkel et al., 2022), suggesting that they are more likely to view sibling relationships as warm and close. Hence, it is also necessary to focus on the moderating effect of siblings’ birth order when exploring the mechanism of marital and sibling relationships on adolescent development.

In addition, compared with studies on the Caucasian sample, sibling research have been underrepresented under the Chinese background for over 30 years because of the one-child policy (Chen and Shi, 2017). The recent adoption of the two-child policy in 2016 represented a major change in social policy that has influenced individual families’ structures and dynamics (Wang et al., 2021). Historically, siblings have been considered a critical part of individuals’ lives in China because of the Confucian values of family duty and solidarity. They are generally called *shou zu*, with the literal meaning being hand and foot relationships, indicating the prominence of siblings’ role as a natural and essential part of one’s body (Wang et al., 2022). Chinese families hold stronger values for family harmony and cohesion; thus, first-born siblings shoulder more family obligations and earn the utmost respect from later-born siblings. Adolescents in the East have been found to be more likely to seek support and companionship from siblings, whereas those in the West more often turn to friends for support (French et al., 2001; Zhang et al., 2023). Hence, it is worth exploring the effect of sibling relationships within contemporary Chinese culture, especially because the number of families with more than one child is growing in China.

**FIGURE 1:**
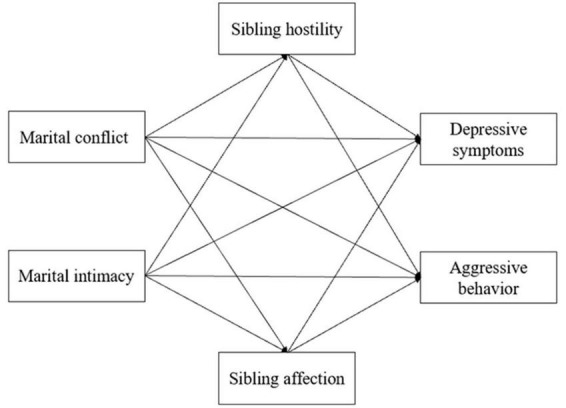
The hypothesized mediation model.

Therefore, guided by family system theory, the primary objective of the present study was to articulate a theoretical framework to investigate the impact of marital relationships on adolescent behavioral problems while examining the mediating role of sibling relationships in multichild families in China (see Figure 1). We hypothesized that marital conflict/intimacy associate adolescent depressive symptoms and aggressive behavior separately and that sibling hostility/affection mediate the above associations after controlling for covariates. In addition, we also explored whether the estimated parameters of the mediation model differ by birth order and hypothesized that first-born and second-born adolescents perform differently. While the study design does not facilitate temporal or causal inferences, it holds the potential to elucidate the association between marital relationships and adolescent development. Additionally, it can provide evidence-based support for interventions addressing behavioral problems during adolescence.

## 2 Materials and methods

### 2.1 Procedure and participants

The current study recruited students from 6 elementary schools and 6 junior high schools in three cities of Henan Province located in central China. The study obtained approval from the Research Ethics Committee at our university. Prior to the investigation, adolescents and their parents submitted signed informed consent forms. Participation was voluntary, allowing participants the flexibility to withdraw at any time. Trained researchers conducted assessments to ensure a standardized data collection process.

We collected a total of 1560 questionnaires. Because the present study mainly focused on sibling relationships, only 892 adolescents from two-child families were chosen. In addition, we were interested in the mother-father dyad; thus, 50 adolescents without a participating parent were excluded. The final analytic sample consisted of 842 adolescents. Among the 842 participants, 389 (46.2%) were boys, 439 (52.1%) were girls, and 14 (1.7%) did not report their sex. The mean age was 12.60 years (*SD* = 1.48), with a mean age gap of 7.14 years (*SD* = 3.55). In addition, 460 (54.6%) adolescents were first-born, 372 (44.2%) adolescents were second-born, and 10 (1.2%) adolescents did not report their birth order.

The mean ages of the participants’ mothers and fathers were 40.74 years (*SD* = 5.58) and 42.12 years (*SD* = 4.68), respectively. Regarding parental education, 418 (51.7%) mothers and 382 (47.7%) fathers had a junior secondary education or lower. A total of 244 (30.3%) mothers and 110 fathers (13.9%) were unemployed. In addition, for 487 (61.0%) families, the average monthly household income was less than 5,000 yuan.

### 2.2 Measures

#### 2.2.1 Marital relationship

Marital relationship was measured with the Parental Relationship Inventory (PRI; Dong and Lin, 2011) revised by the “National Children’s Study of China (NCSC).” It includes two dimensions, i.e., marital intimacy (5 items, e.g., “My parents care about each other.”) and marital conflict (10 items, e.g., “My parents often blame each other and hold different opinions on things”). This inventory asks students to report their parents’ daily activities on a 5-point Likert scale ranging from 1 (describes very poorly) to 5 (describes very well), and a higher score on this scale indicates higher relationship intimacy or conflict. The Cronbach’s α values were 0.85 for marital intimacy and 0.90 for marital conflict in this study.

#### 2.2.2 Sibling relationship

The Sibling Relationship Inventory (SRI; Boer et al., 1997) was adopted to measure sibling relationships. This inventory requires adolescents to report the frequency of various sibling behaviors. The subscales of sibling affection (8 items, e.g., “How often do you take care of your (target) siblings when the adults aren’t around?”) and sibling hostility (5 items, e.g., “How often do you feel mad or angry at your (target) siblings?”) were used in this study. The adolescents responded on a 5-point Likert scale (1 = never to 5 = always). A higher score indicates higher levels of relationship positivity or hostility. In the current study, the Cronbach’s α values were 0.79 and 0.70 for sibling affection and sibling hostility, respectively.

#### 2.2.3 Depressive symptoms

Depressive symptoms were assessed with the Children’s Depression Inventory-Short Version (CDI-S; Kovacs, 1992). The 10 items in the CDI-S cover “sadness,” “pessimism,” “self-deprecation,” “self-hate,” “crying spells,” “irritability,” “negative body image,” “loneliness,” “lack of friends,” and “feeling unloved” (e.g., “I don’t know if anyone loves me”). The adolescents were asked to rate the degree to which each statement applied to them during the past 2 weeks on a scale from 0 to 2. The total scores range from 0 to 20, with higher scores indicating more severe depressive symptoms. In the current study, the Cronbach’s α was 0.80.

#### 2.2.4 Aggressive behavior

The 9-item Physical Aggressive Behavior subscale of the Bush-Perry Aggressive Behavior Questionnaire (BPAQ; Buss and Perry, 1992) was used. A sample item is “Given enough provocation, I may hit another person.” The items were answered on a five-point Likert scale ranging from 1 (almost never true) to 5 (almost always true), and higher scores indicated more severe aggressive behavior. The Cronbach’s α was 0.76 in this study.

### 2.3 Covariates

The control variables include adolescent age, sex and family socioeconomic status (SES). To calculate family SES, family income, parents’ occupation, and educational level were transferred into Z scores and added up to composite family SES (Zhang et al., 2020).

### 2.4 Statistical analysis

First, we performed descriptive statistical analysis and examined the correlations between variables using SPSS 26.0. Then, we generated a direct effects model of marital conflict/intimacy on depressive symptoms/aggressive behavior. Next, we examined the mediating role of sibling affection and sibling hostility using Mplus 8.0. The bootstrap method was used to examine the statistical significance of the mediation effects, which generated an approximation of the sampling distribution to obtain confidence intervals for an indirect effect.

Furthermore, we conducted multiple group analysis (MGA) to examine the moderating role of birth order. The differences in the Chi square statistic were used to compare nested models with an iterative, stepwise approach (Bowen and Guo, 2012). Specifically, all model paths were initially estimated separately for first-born and second-born groups. If two separate models fit the data well, an unconstrained model with all coefficients freely estimated was then compared to a constrained model with all coefficients equaled between groups. If there was a significant change in the chi-square statistic between the unconstrained model and constrained model, it indicated that birth order played a moderating role. Then, each path was constrained to be identical across two groups to test which direct and indirect model paths differed by birth order.

## 3 Results

### 3.1 Descriptive statistics

Table 1 shows the mean, standard deviation, skewness, kurtosis statistics and correlations for all continuous variables. The skewness and kurtosis values were mostly within an acceptable range (i.e., skewness < |2.0| and kurtosis < |7.0|; Curran et al., 1996). There were significant correlations between all variables. Specifically, marital intimacy was negatively correlated with sibling hostility, depressive symptoms and aggressive behavior but positively correlated with sibling affection. Marital conflict was positively correlated with sibling hostility, depressive symptoms and aggressive behavior but negatively correlated with sibling affection. Sibling affection was negatively correlated with depressive symptoms and aggressive behavior, while sibling hostility was positively correlated with them.

**TABLE 1:** Descriptive statistics and correlational analysis (*N* = 842).

Variables	M ± SD	Skewness	Kurtosis	1	2	3	4	5	6
1 Marital intimacy	3.82 ± 0.89	-0.56	-0.29	1					
2 Marital conflict	2.16 ± 0.93	0.92	0.30	-0.49**	1				
3 Sibling affection	3.58 ± 0.72	-0.41	-0.07	0.31**	-0.16**	1			
4 Sibling hostility	1.81 ± 0.62	1.17	2.18	-0.24**	0.33**	-0.27**	1		
5 Depressive symptoms	3.31 ± 3.23	1.35	1.64	-0.21**	0.25**	-0.16**	0.20**	1	
6 Aggressive behavior	2.29 ± 0.71	0.37	-0.15	-0.18**	0.32**	-0.20**	0.35**	0.24**	1

**p < 0.01.

### 3.2 Mediating effects of sibling relationship

First, we established a direct effects model with paths from marital intimacy and marital conflict to depressive symptoms and aggressive behavior. Because of the high correlation between marital conflict and marital intimacy, we established a correlational path between them to avoid type I errors. The path analysis showed that marital conflict was directly and positively associated with depressive symptoms (β = 0.19, *p* < 0.001) and aggressive behavior (β = 0.31, *p* < 0.001). Marital intimacy was directly and negatively associated with depressive symptoms (β = −0.12, *p* < 0.01), but the association between marital intimacy and aggressive behavior was not significant.

**FIGURE 2:**
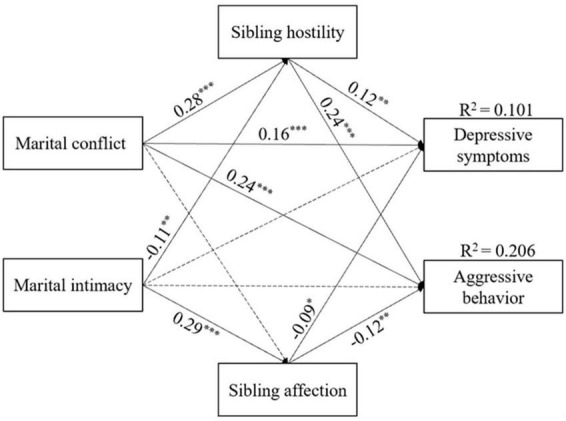
Standardized regression coefficients of the mediation model among marital conflict, marital intimacy, sibling hostility, sibling affection, adolescent depressive symptoms and aggressive behavior. *p < 0.05; **p < 0.01; and ***p < 0.001.

Based on the direct effects model, we inserted sibling affection and sibling hostility as mediators of the relationship between marital conflict/intimacy and depressive symptoms/aggressive behavior. Non-significant control variables were removed to find the optimal model. The results showed that this model fit well, χ^2^ (13) = 3.09, *p* < 0.001; *CFI* = 0.947; *RMSEA* = 0.050; *SRMR* = 0.044, and accounted for 10.1% of the variance in depressive symptoms and 20.6% of the variance in aggressive behavior. As shown in Figure 2, more marital conflict was linked to more sibling hostility (β = 0.28, *p* < 0.001), more aggressive behavior (β = 0.24, *p* < 0.001), and more depressive symptoms (β = 0.16, *p* < 0.001); more marital intimacy was associated with more sibling affection (β = 0.29, *p* < 0.001) and less sibling hostility (β = −0.11, *p* < 0.01). Sibling hostility was positively related to depressive symptoms (β = 0.12, *p* < 0.01) and aggressive behavior (β = 0.24, *p* < 0.001), while sibling affection was negatively related to depressive symptoms (β = −0.09, *p* < 0.05) and aggressive behavior (β = −0.12, *p* < 0.01).

Then, we performed the bootstrapping method to calculate the indirect effects. Path analysis identified 6 significant paths: from marital conflict to adolescent depressive symptoms and aggressive behavior through sibling hostility and from marital intimacy to adolescent depressive symptoms and aggressive behavior through both sibling hostility and sibling affection. More detailed results are shown in Table 2. These findings revealed that both marital conflict and marital intimacy were related to adolescent depressive symptoms and aggressive behavior, and their relationships were mediated by sibling hostility and sibling affection.

**TABLE 2:** Bias-corrected bootstrap test of the mediating effect of the model.

Path	Standardized indirect effect values	Effect size	95% confidence interval	
			Lower	Upper
MC→SH→DS	**0**.**112**	**25**.**34%**	**0**.**036**	**0**.**199**
MC→SH→AB	**0**.**051**	**11**.**54%**	**0**.**031**	**0**.**075**
MC→SA→DS	-0.004	0.90%	-0.039	0.025
MC→SA→AB	-0.001	0.23%	-0.011	0.007
MI→SH→DS	-**0**.**045**	**10**.**18%**	-**0**.**102**	-**0**.**005**
MI→SH→AB	-**0**.**021**	**4**.**75%**	-**0**.**039**	-**0**.**004**
MI→SA→DS	-**0**.**093**	**21**.**04%**	-**0**.**186**	-**0**.**008**
MI→SA→AB	-**0**.**028**	**6**.**33%**	-**0**.**048**	-**0**.**011**

MC, marital conflict; MI, marital intimacy; SH, sibling hostility; SA, sibling affection; DS, depressive symptoms; AB, aggressive behavior. Bold values mean the result is significant.

### 3.3 Moderating effects of birth order

**FIGURE 3:**
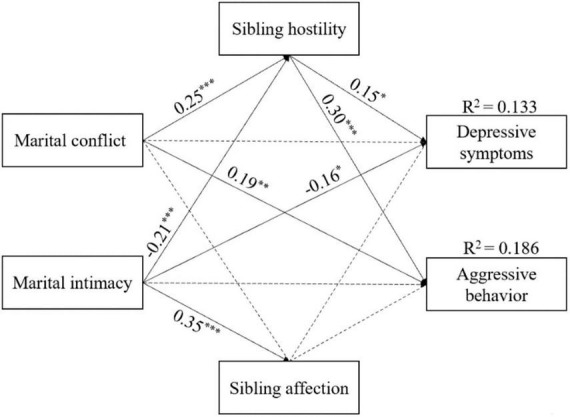
Standardized regression coefficients of the mediation model among marital conflict, marital intimacy, sibling hostility, sibling affection, adolescent depressive symptoms and aggressive behavior in first-born adolescents. *p < 0.05; **p < 0.01; ***p < 0.001.

**FIGURE 4:**
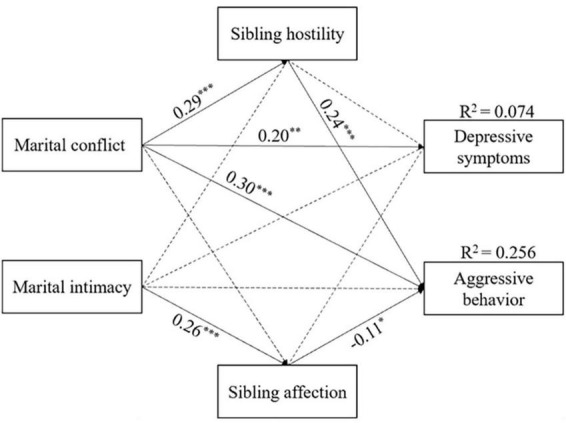
Standardized regression coefficients of the mediation model among marital conflict, marital intimacy, sibling hostility, sibling affection, adolescent depressive symptoms and aggressive behavior in second-born adolescents. *p < 0.05; **p < 0.01; ***p < 0.001.

To address our research question regarding the moderating influence of birth order, we employed a multiple-group path model to examine potential differences between first-born and second-born adolescents. The results revealed a good fit for both the first-born model [χ^2^ (13) = 3.02, *p* < 0.001; *CFI* = 0.919; *RMSEA* = 0.067; *SRMR* = 0.062] and the second-born model [χ^2^ (13) = 1.13, *p* < 0.001; *CFI* = 0.992; *RMSEA* = 0.019; *SRMR* = 0.033]. Figures 3, 4 present the standardized results of the models for first-born and second-born adolescents, respectively.

Then the unconstrained model [χ^2^ (26) = 2.07, *p* < 0.001; *CFI* = 0.947; *RMSEA* = 0.051; *SRMR* = 0.051] and constrained model [χ^2^ (38) = 2.01, *p* < 0.001; *CFI* = 0.927; *RMSEA* = 0.050; *SRMR* = 0.059] were established. A notable change in the chi-square value was observed [Δχ^2^ (12) = 1.88, *p* < 0.05]. Consequently, the unconstrained model was selected as the baseline. Next, each path was constrained to be equal between first-born and second-born adolescents to test the differences in model paths. There was a significant difference from the baseline model if χ^2^ values were higher than 3.84 (*df* = 1) or 5.99 (*df* = 2).

The results indicated that the negative association between marital intimacy and sibling hostility was significantly stronger in first-born adolescents [Δχ^2^ (1) = 6.06]. The indirect effect of marital relationship on adolescent problem behaviors through sibling relationships also differed between first-born and second-born adolescents. Specifically, the association between marital intimacy and depressive symptoms through sibling hostility was significantly stronger in first-born adolescents [Δχ^2^ (2) = 6.28], as was the association between marital intimacy and aggressive behavior through sibling hostility [Δχ^2^ (2) = 6.95]. More detailed results are shown in Table 3.

**TABLE 3:** Path analysis testing a multiple-group path model.

Pathways	Frist-born			Second-born			Test of birth order difference	
	Est	SE	*p*	Est	SE	*p*	Δχ^2^ (Δ*df*)	*p*
**Direct paths**								
MI→SA	0.35	0.06	<0.001	0.26	0.06	<0.001	0.98 (1)	
MI→SH	-**0**.**21**	**0**.**05**	<**0**.**001**	-**0**.**02**	**0**.**06**	>**0**.**05**	**6.06 (1)**	<**0**.**05**
MI→DS	-0.16	0.06	<0.05	0.00	0.06	>0.05	3.65 (1)	
MI→AB	0.09	0.06	>0.05	-0.02	0.06	>0.05	2.52 (1)	
MC→SA	0.05	0.06	>0.05	0.01	0.06	>0.05	0.25 (1)	
MC→SH	0.25	0.05	<0.001	0.29	0.06	<0.001	0.26 (1)	
MC→DS	0.09	0.06	>0.05	0.20	0.06	<0.01	2.02 (1)	
MC→AB	0.19	0.06	<0.01	0.30	0.06	<0.001	2.11 (1)	
SA→DS	-0.09	0.06	>0.05	-0.03	0.06	>0.05	0.58 (1)	
SA→AB	-0.09	0.05	>0.05	-0.11	0.05	<0.05	0.03 (1)	
SH→DS	0.15	0.06	<0.05	0.11	0.06	>0.05	0.22 (1)	
SH→AB	0.30	0.06	<0.001	0.24	0.05	<0.001	0.88 (1)	
**Indirect paths**								
MI→SA→DS	-0.11	0.08	>0.05	-0.03	0.05	>0.05	1.56 (2)	
MC→SA→DS	-0.02	0.03	>0.05	-0.00	0.02	>0.05	0.83 (2)	
MI→SH→DS	-**0**.**11**	**0**.**06**	<**0**.**05**	-**0**.**01**	**0**.**03**	>**0**.**05**	**6.28 (2)**	<**0**.**05**
MC→SH→DS	0.13	0.06	<0.05	0.11	0.07	>0.05	0.48 (2)	
MI→SA→AB	-0.03	0.01	>0.05	-0.02	0.01	>0.05	1.01 (2)	
MC→SA→AB	-0.00	0.01	>0.05	-0.00	0.01	>0.05	0.28 (2)	
MI→SH→AB	-**0**.**05**	**0**.**02**	<**0**.**01**	-**0**.**01**	**0**.**01**	>**0**.**05**	**6.95 (2)**	<**0**.**05**
MC→SH→AB	0.06	0.02	<0.01	0.05	0.01	<0.001	1.14 (2)	

MC, marital conflict; MI, marital intimacy; SH, sibling hostility; SA, sibling affection; DS, depressive symptoms; AB, aggressive behavior. Bold values mean the result is significant.

## 4 Discussion

A substantial number of studies have examined the impact of marital conflict on the negative function of adolescents while ignoring the role of marital intimacy (Erath et al., 2006; Philbrook et al., 2018; Swerbenski et al., 2023). Based on family system theory, we extended previous studies by including both marital conflict and intimacy, as well as depressive symptoms and aggressive behavior, in an integrated model. Additionally, we examined the mediating role of sibling relationships and the moderating influence of birth order within the context of the two-child policy in China. This not only enriches our understanding of multichild families but also contributes to a more comprehensive exploration of familial dynamics in the Chinese culture.

Aligned with previous findings, we found that marital conflict was positively associated with adolescent behavioral problems, while marital intimacy was negatively associated with them. Consistent with social learning theory, adolescents constantly learn how to deal with contradictions in the context of marital conflict and unconsciously develop the psychological tendency of aggression (Erath et al., 2006). Conversely, marital intimacy guides adolescents to cope with difficulties in proper ways rather than using violence, thus decreasing their aggressive behaviors (Herman and McHale, 1993). The emotional security hypothesis attributes such influence to adolescents’ sense of emotional security (Cummings et al., 2012). An open and positive marital relationship can provide emotional support, a source of delight and purpose in life for adolescents, which are vital to their mental health and psychosocial adjustment (Li et al., 2020; Zhang et al., 2022). In contrast, exposure to marital conflict may evoke adolescents’ fear, pain, vigilance and hostility, potentially leading to depression and other mental illnesses (Cummings et al., 2012; Swerbenski et al., 2023). Thus, fostering a harmonious marital relationship may serve as a pivotal factor in mitigating adolescent depressive symptoms and aggressive behaviors.

The results of mediation analyses showed that marital conflict is indirectly associated with adolescent depressive symptoms and aggressive behavior through sibling hostility. This connection could be explained in two ways. First, adolescents who frequently view their parents quarrel are likely to imitate this behavior in interactions with others, such as siblings (Erath et al., 2006; van Berkel et al., 2022). Second, adolescents may be forced to choose a side when parents fight (Poortman and Voorpostel, 2009), which is likely to stress sibling interactions as well. Sibling hostility, in turn, correlates with later behavioral problems. Drawing upon Patterson’s coercive cycle model, negative sibling interaction may contribute to negative reinforcement of externalizing problems, thereby resulting in aggressive behavior (Patterson, 1984). Sibling hostility can also give rise to internalizing adjustment problems through decreased emotional support (Kim et al., 2007). In contrast to the mediational role of sibling hostility, the results showed that marital conflict is not related to adolescent behavioral problems through sibling affection. This lack of relationship may be due to the fact that sibling affection, as a positive interaction between siblings, is more likely to be influenced by marital intimacy. Marital conflict, on the other hand, had a smaller effect on positive interactions but was associated with sibling hostility by triggering socially learned behaviors (Poortman and Voorpostel, 2009; Chen, 2023). Above all, marital conflict has implications for sibling hostility, and with high sibling hostility, adolescents are at much greater risk of depressive symptoms and aggressive behavior.

In addition to testing our hypothetical mediation model, this study also presented a research question about the moderating role of siblings’ birth order. Through a comprehensive examination of all pathways, we found that the pathway from marital intimacy to sibling hostility, as well as the indirect pathway from marital intimacy to depressive symptoms and aggressive behavior through sibling hostility, were stronger in first-born adolescents, as compared with second-born adolescents. That is, first-born adolescents are potentially more sensitive to marital intimacy than second-born adolescents. In fact, it is common for parental comparisons and favoritism to occur after the birth of younger siblings in multichild families, especially in Asian cultures (Chen et al., 2021; Zhao et al., 2021). As a form of negative parenting, parental favoritism has implications for adolescent emotional and behavioral problems (Jensen and Whiteman, 2014). Subsequently, eldest children who lose parents’ attention tend to exhibit sibling hostility, depressive symptoms and aggressive behavior (Buhrmester and Furman, 1990; Chen, 2023). Given this, parents should establish a family atmosphere that is more intimate to protect their first child’s mental health.

There are some limitations to the current study. First, this research employed a cross-sectional design, indicating that the results are only correlational and not causal. The associations among the variables and possible mediation and moderation that should be further investigated in prospective longitudinal studies. Second, the information was assessed with self-report questionnaires among adolescents and parental perceptions of their relationships were not assessed. Future researchers can obtain paired data from parents and adolescents to compare the effects of various subjects. Last, this population was from 12 elementary and junior high schools in one province, the sample was relatively homogenous, which may limit the external validity of the results, future studies could include more representative samples.

Despite its limitations, this study has important theoretical and practical implications. Theoretically, it provided an empirical framework to examine the associations between marital relationships and adolescent behavioral problems, as well as the mediating role of sibling relationships and the moderating effect of birth order. This research also extended previous studies by including two dimensions of parental relationships and sibling relationships, which contributes to more nuanced understanding of interactions between family subsystems. From a practical perspective, the results indicated that preventions aimed at reducing adolescent depressive symptoms and aggressive behaviors should also tackle family relationships, such as parental relationships and sibling relationships.

## 5 Conclusion

In conclusion, based on family system theory, the current study comprehensively investigated the mechanism by which marital and sibling subsystems affect adolescent development. The results revealed that marital intimacy was associated with adolescent behavioral problems through sibling affection and sibling hostility. While marital conflict was associated with sibling hostility, which was then further associated with adolescents behavioral problems. Additionally, we found a moderating role of birth order, that is first-born adolescents were more sensitive to marital relationship.

## Data availability statement

The raw data supporting the conclusions of this article will be made available by the authors, without undue reservation.

## Ethics statement

The studies involving humans were approved by the Ethical Committee of Collaborative Innovation Center of Assessment toward Basic Education Quality. The studies were conducted in accordance with the local legislation and institutional requirements. Written informed consent for participation in this study was provided by the participants’ legal guardians/next of kin.

## Author contributions

ZL: Conceptualization, Methodology, Writing – original draft, Writing – review and editing, Formal analysis, Investigation. YZ: Methodology, Writing – review and editing. RH: Writing – review and editing. RL: Writing – review and editing. YL: Writing – review and editing. ZY: Writing – review and editing. MQ: Writing – review and editing. FC: Supervision, Writing – review and editing, Resources, Validation.
